# Regulation of the p19^Arf^/p53 pathway by histone acetylation underlies neural stem cell behavior in senescence-prone SAMP8 mice

**DOI:** 10.1111/acel.12328

**Published:** 2015-02-26

**Authors:** Raúl Soriano-Cantón, Ana Perez-Villalba, José Manuel Morante-Redolat, María Ángeles Marqués-Torrejón, Mercé Pallás, Francisco Pérez-Sánchez, Isabel Fariñas

**Affiliations:** 1 Centro de Investigación Biomédica en Red en Enfermedades Neurodegenerativas (CIBERNED), Universidad de ValenciaBurjassot, 46100, Spain; 2 Departamento de Biología Celular, Universidad de ValenciaBurjassot, 46100, Spain; 3 Departamento de Farmacología y Química Terapéutica, Instituto de Biomedicina de la Universidad de BarcelonaBarcelona, 08028, Spain

**Keywords:** aging, adult neurogenesis, histone deacetylases, histone acetyltransferases, stem cell niche, SAMP8 mice

## Abstract

Brain aging is associated with increased neurodegeneration and reduced neurogenesis. B1/neural stem cells (B1-NSCs) of the mouse subependymal zone (SEZ) support the ongoing production of olfactory bulb interneurons, but their neurogenic potential is progressively reduced as mice age. Although age-related changes in B1-NSCs may result from increased expression of tumor suppressor proteins, accumulation of DNA damage, metabolic alterations, and microenvironmental or systemic changes, the ultimate causes remain unclear. Senescence-accelerated-prone mice (SAMP8) relative to senescence-accelerated-resistant mice (SAMR1) exhibit signs of hastened senescence and can be used as a model for the study of aging. We have found that the B1-NSC compartment is transiently expanded in young SAMP8 relative to SAMR1 mice, resulting in disturbed cytoarchitecture of the SEZ, B1-NSC hyperproliferation, and higher yields of primary neurospheres. These unusual features are, however, accompanied by premature loss of B1-NSCs. Moreover, SAMP8 neurospheres lack self-renewal and enter p53-dependent senescence after only two passages. Interestingly, *in vitro* senescence of SAMP8 cells could be prevented by inhibition of histone acetyltransferases and mimicked in SAMR1 cells by inhibition of histone deacetylases (HDAC). Our data indicate that expression of the tumor suppressor p19, but not of p16, is increased in SAMP8 neurospheres, as well as in SAMR1 neurospheres upon HDAC inhibition, and suggest that the SAMP8 phenotype may, at least in part, be due to changes in chromatin status. Interestingly, acute HDAC inhibition *in vivo* resulted in changes in the SEZ of SAMR1 mice that resembled those found in young SAMP8 mice.

## Introduction

The ‘stem cell aging’ hypothesis suggests that loss of potential in somatic stem cell pools underlies the progressive decline in tissue renewal observed in multiple systems during aging (van Deursen, 2014). Lifelong generation of olfactory bulb (OB) interneurons takes place in the subependymal zone (SEZ; also known as ventricular/subventricular zone or V/SVZ) of the lateral ventricle wall and is supported by a population of neural stem cells (NSCs, also known as B1 cells). B1 cells divide slowly and can hence retain exogenously administered traceable nucleosides for several weeks, generating transit-amplifying progenitor (TAP) cells, which in turn give rise to neuroblasts that migrate anteriorly to the OB where they differentiate into granular and periglomerular interneurons (Silva-Vargas *et al*., 2013).

Reduced levels of olfactory neurogenesis that are primarily due to decreased numbers of stem-like cells are found in elderly animals (Maslov *et al*., 2004; Luo *et al*., 2006; Molofsky *et al*., 2006; Shook *et al*., 2012). Many NSCs remaining in the aged SEZ are primarily quiescent; they are less likely to undergo cellular division or neuronal differentiation than NSCs in the young adult brain, with lower chances of survival (Ahlenius *et al*., 2009). Those cells remaining in cell cycle, however, are more proliferative than young NSCs, due to increased rates of cell cycle reentry, which can lead to transient expansion of cells before terminal differentiation (Stoll *et al*., 2011). NSC permanent cell cycle exit results in their differentiation into S100β^+^ astrocyte-like cells (Raponi *et al*., 2007; Porlan *et al*., 2013), although it has been reported that a fraction of B1 cells may also integrate within the aged ependymal cell layer and acquire ependymal features (Luo *et al*., 2008).

Although age-related declines in stem cell function have been associated with altered expression of some growth factors (Tropepe *et al*., 1997; Enwere *et al*., 2004), tumor suppressors p16^Ink4a^ and p19^Arf^ (hereafter p16 and p19; encoded by the *Cdkn2a* or *Ink4a/Arf* locus by the use of two different promoters and alternative reading frames) also appear to play a role (Molofsky *et al*., 2006; Ahlenius *et al*., 2009; Mikheev *et al*., 2009). Cell cycle inhibitor p16 binds to and inhibits CDK4 and CDK6 to retain the growth-suppressive activity of Rb family proteins, whereas p19 binds to and antagonizes the activity of the ubiquitin ligase Mdm2, thereby stabilizing and activating p53 (Gil & Peters, 2006). Histone acetylation controlled by the antagonistic actions of histone acetyltransferases (HATs) and histone deacetylases (HDACs) which either favor or repress transcription (Willis-Martinez *et al*., 2010) has been involved in the regulation of this locus in other cells (Matheu *et al*., 2005; Simboeck *et al*., 2011).

Effects of aging in neural dysfunction have been analyzed in several strains of senescence-accelerated mice (SAM) originated from inbreeding and selection for the early appearance of aging features (Takeda, 2009). Relative to control senescence-resistant SAMR1 mice, young senescence-prone SAMP8 mice exhibit neuronal loss, gliosis, and progressive cognitive deficits (Takeda, 2009; Tomobe & Nomura, 2009). Proliferation in the SEZ and in the dentate gyrus subgranular zone (SGZ), another neurogenic niche, is increased in 2-month-old (2-m) but reduced in 10-m SAMP8 mice (Gang *et al*., 2011; Díaz-Moreno *et al*., 2013). Moreover, although more neuroblasts are produced in young SAMP8 mice, they are lost at a higher rate due to apoptosis (Gang *et al*., 2011). These results have been interpreted as a reaction to the neuronal dysfunction/degeneration observed in these animals, but they also raise the possibility that the senescent-prone genetic background has a direct impact on intrinsic properties of NSCs. We have found that increased loss of NSCs in 10-m SAMP8 mice is preceded by their transient overactivation. Despite the higher proliferative activity of young SAMP8 NSCs, they expressed increased levels of p19 and p53 and entered senescence prematurely when cultured *in vitro*, a deficit that could be restored by abrogation of p53 activity and, interestingly, by inhibition of the activity of HATs. Moreover, we could induce a senescent phenotype, together with increases in p19 and p53, by *in vitro* treatment with inhibitors of HDACs, whereas *in vivo* treatment resulted in morphological changes of the SEZ resembling those of young SAMP8 mice.

## Results

### Increased proliferation and abnormal positioning of B1 NSCs in SAMP8 mice precedes their exhaustion

**Fig 1:**
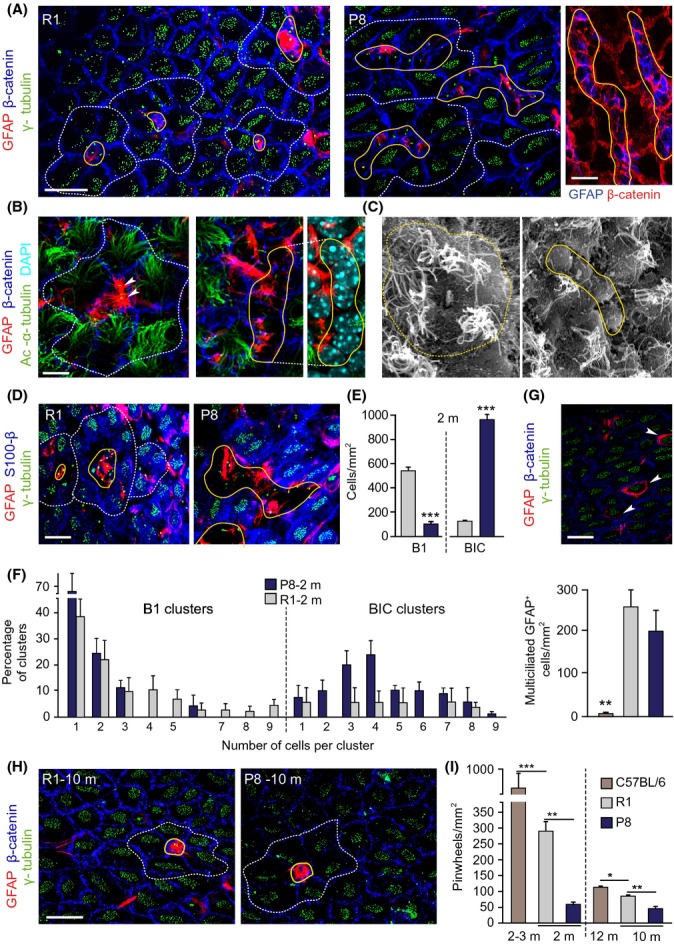
The cytoarchitecture of the SEZ is altered in young P8 mice. (A) Whole-mounts from 2-m SAMR1 (R1) and SAMP8 (P8) mice immunostained for GFAP, β-catenin, and γ-tubulin. (B) GFAP, β-catenin, and acetylated α-tubulin. Arrowheads point to B1-cell primary cilium. (C) Whole-mounts in scanning electron microscopy. Note the presence of misshapen pinwheels with abutted GFAP cells in P8 mice. (D) GFAP, γ-tubulin, and S100β in R1 (left) and P8 (right) whole-mounts. BICs are S100β^−^. (E) Number of B1 cells and BICs in SEZs at 2-m. (F) Distribution of GFAP^+^ B1 cells (left) or BICs (right) at 2-m. (G) GFAP, γ-tubulin, and S100β in whole-mounts showing GFAP^+^ multiciliated cells (white arrowheads) in the plane of the ependyma (above) and quantification of these cells (below) in 2-m R1, P8, and C57BL/6 mice. (H) GFAP, γ-tubulin, and β-catenin in SEZ whole-mounts at 10-m. Only normally organized pinwheels are observed in both genotypes. (I) Pinwheel density in the dorsal SEZ of 2- and 10-m R1 and P8 mice, and in 3- and 12-m C57BL/6 mice. Pinwheels are delineated by dashed white lines, and B1 cells/BICs are delineated by solid yellow lines. Data are shown as mean ± SEM of the indicated number of mice (*n*) from each strain (*P <* *0.05; **P <* *0.01; ***P <* *0.001). Scale bars: (A, left) 10 μm; (B) 5 μm; in (A, right), (D), (G) and (H) 20 μm.

A previous analysis had indicated a transient increase in BrdU incorporation in the SEZ of young SAMP8 mice (Díaz-Moreno *et al*., 2013). To focus specifically on B1 NSCs, we analyzed whole-mount preparations of the lateral ventricle wall by confocal microscopy. The ventricle wall surface is characterized by the repetition of ‘pinwheel’ units, arrangements in which the uniciliated apical thin processes of one or several GFAP^+^ B1 NSCs are encircled by multiciliated ependymal cells (Mirzadeh *et al*., 2008). Immunostaining for GFAP, β-catenin to delineate cell membranes, and either γ-tubulin as a marker of cilial basal bodies or acetylated α-tubulin to label cilial cytoskeleton, revealed a normal pinwheel organization in SAMR1 mice (Fig.1A,B). Compared to other mouse strains, however, 2-m SAMR1 mice had fewer B1 apical surfaces (543 ± 34 *per* mm^2^ of dorsal SEZ, *n* = 3, *vs*. 1,732 ± 53 in C57BL/6 mice, *n* = 3) and pinwheels (289 ± 30 *per* mm^2^, *n* = 4, *vs*. 952 ± 45 in C57BL/6 mice, *n* = 3) (for CD1 mice, see also Mirzadeh *et al*., 2008; Shook *et al*., 2012), suggesting potential effects of the SAM genetic background in B1-cell population size.

Although all pinwheel components could also be recognized in 2-m SAMP8 mice, the organization was altered (Fig.1A,B). The number of pinwheels with thin B1-cell processes at their core was remarkably low (59 ± 7 *per* mm^2^, *n* = 3). In contrast, we could observe misshapen pinwheels formed by ependymocytes surrounding various GFAP^+^ cells with large apical areas in the ependymal layer plane, a feature that was also evident in analyses with scanning electron microscopy (Fig.1A–C). Because cells in these groups were uniciliated and GFAP^+^ but did not express the calcium-binding protein S100β, a marker of ependymal cells and terminally differentiated astrocytes (Raponi *et al*., 2007) (Fig.1D), we named them ‘B1-like interposed cells’ (BICs). BICs were very frequent in SAMP8 mice compared to the normally looking B1 cells; in addition, most BICs were arranged in clusters of 3–8 cells (Fig.1E, F), suggesting that GFAP^+^ cells were increased.

We also detected high numbers of cuboidal GFAP^+^ cells with multiple cilia integrated within the ependymal layer (Fig.1G). This type of cell reportedly increases during aging but is rarely found in young C57BL/6 and CD1 mice (Luo *et al*., 2008; Capilla-Gonzalez *et al*., 2014). Indeed, we could find only 7 ± 3 cells *per* mm^2^ (*n* = 3) in the dorsal SEZ of 2-m C57BL/6 mice, but 49 ± 21 cells *per* mm^2^ at 12-m and 52 ± 6 at 24-m (*n* = 2 each). Young SAMR1 and SAMP8 mice had similarly elevated proportions of these cells (Fig.1G), suggesting that this feature could have been selected through inbreeding.

Notably, the appearance of the SEZ of 10-m SAMP8 mice was similar to that found in SAMR1 controls at 10-m and in normal mouse strains at 12-m. Quantification of pinwheel density revealed a significant reduction in SAMR1 mice between 2 and 10 months, a period at which most age-related dysfunctions develop in SAM strains (Tomobe & Nomura, 2009), similar to reductions observed in C57BL/6 mice. In 10-m SAMP8 mice, however, pinwheels were significantly less frequent (Fig.1H,I), indicating that BICs were a transient feature and that loss of NSCs is accelerated in SAMP8 mice.

**Fig 2:**
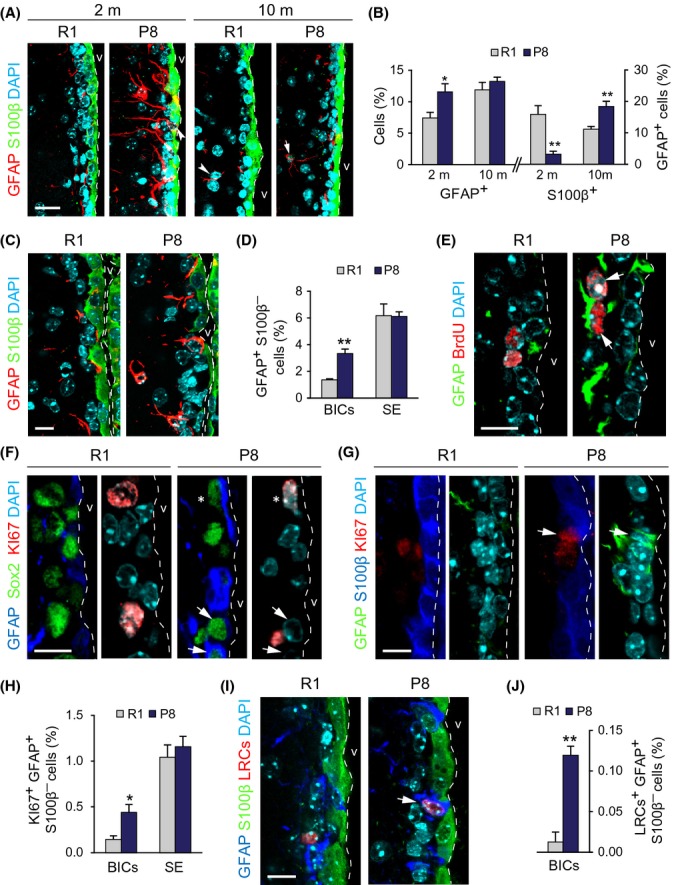
Increased number of proliferating NSCs in the SEZ of young P8 mice. (A) GFAP and S100β detection in the SEZs of 2- and 10-m R1 and P8 mice. (B) Percentage of GFAP^+^/DAPI cells (left) and of GFAP^+^S100β^+^/GFAP^+^ cells (right) in the SEZs of 2- and 10-m R1 and P8 mice. (C) GFAP and S100β in the SEZ of 2-m R1 and P8 mice. (D) Percentage of GFAP^+^S100β^−^ BICs and subependymal (SE) cells relative to total cells. (E) GFAP and BrdU in the SEZ of 2-m R1 and P8 mice. (F) GFAP, Sox2, and Ki67 in the SEZ of 2-m R1 and P8 mice. (G) GFAP, S100β, and Ki67 in the SEZ of 2-m R1 and P8 mice. (H) Percentage of Ki67^+^GFAP^+^S100β^─^ BICs and subependymal (SE) cells relative to total cells. (I) GFAP, S100β, and LRCs in the SEZ of 2-m R1 and P8 mice. (J) Percentage of GFAP^+^S100β^─^ BrdU^+^ label retaining BICs. Data are shown as mean ± SEM of 3 independent mice from each strain (*P <* *0.05; **P <* *0.01). Dashed white line indicates the lateral ventricle (v) limit. White arrowheads point at single-positive cells and white arrows at doubly positive cells. Asterisks indicate triple-positive cells. Scale bars: 10 μm.

In SEZ sections, we also observed an overall increase in GFAP, both in the number and intensity of positive cells, in 2-m SAMP8 mice (Fig.2A,B). Moreover, we detected more GFAP^+^Sox2^+^ cells (relative to DAPI: 16.4 ± 1.1, *n* = 4, *vs*. 10.2 ± 0.3%, *n* = 3, in SAMR1 mice; *P *<* *0.01) and more GFAP^+^ cells were also Sox2^+^ and nestin^+^ (23.1 ± 1.3 *vs*. 16.1 ± 1.2% in SAMR1 mice; *n* = 3, *P *<* *0.05), but fewer were S100β^+^ (3.3 ± 0.9% *vs*. 15.9 ± 2.8 in SAMR1 controls; *n* = 4, *P *<* *0.01; Fig.2B). These observations indicated the presence of higher numbers of B1 cells in young SAMP8 mice. Similar to observations in whole-mounts, some GFAP^+^ cells appeared to be integrated within the ependymal layer (Fig.2C). Indeed, the net increase in GFAP^+^ cells found in 2-m SAMP8 mice was due to a selective increase in the number of GFAP^+^S100^β^-BICs, but not subependymal cells (Fig.2C,D). At 10-m, we found similar numbers of GFAP^+^ cells in both genotypes, but significantly more of them were S100β^+^ in SAMP8 mice (Fig.2B), again suggesting that BICs eventually differentiate into S100β^+^ astrocytes and that NSC senescent features develop faster in SAMP8 mice.

### Increased NSC proliferation in young SAMP8 mice is counteracted by cell death of migrating neuroblasts

We next investigated whether more B1 cells in 2-m SAMP8 mice resulted in increased neurogenesis. We did not detect changes in the overall proliferation in the SEZ of SAMP8 mice injected with a single pulse of 5-bromo-2′deoxyuridine (BrdU) one hour before sacrifice (relative to total cells: 8.6 ± 0.6 BrdU^+^ cells *vs*. 9.5 ± 0.5% in SAMR1 mice; *n* = 4). However, the proportions of GFAP^+^BrdU^+^ cells (0.38 ± 0.06% *vs*. a SAMR1 value of 0.22 ± 0.06%; *n* = 4, *P *<* *0.05) and of GFAP^+^Sox2^+^Ki67^+^ cells (1.5 ± 0.1 *vs*. 1.0 ± 0.2%; *n* = 4, *P *<* *0.01) were significantly increased (Fig.2E,F). The increase in Ki67^+^-proliferative GFAP^+^S100β^−^ cells in SAMP8 mice was also specifically due to an increase in Ki67^+^ cells among BICs (relative to total cells: 0.44 ± 0.09 in SAMP8 *vs*. 0.14 ± 0.04% in SAMR1 mice; *n* = 4, *P *<* *0.05; Fig.2G,H), suggesting a correlation between abnormal positioning and proliferation.

Activated B1 cells can incorporate and retain BrdU for several weeks, and we could detect more GFAP^+^ BrdU label retaining cells (BrdU-LRC) in SAMP8 than in SAMR1 mice which had been repeatedly injected with the nucleoside (7 doses in a 12-h period) one month before euthanasia (relative to DAPI: 0.30 ± 0.05 *vs*. 0.16 ± 0.01%, respectively; *n* = 3, *P *<* *0.05). Again, GFAP^+^S100β^−^ LRCs in contact with the ventricle lumen were more frequent in SAMP8 mice (0.12 ± 0.01 *vs*. a SAMR1 value of 0.01 ± 0.01; *n* = 3, *P *<* *0.01; Fig.2I,J).

Activated B1 cells generate proliferating neuroblasts that can be detected with antibodies to doublecortin (DCX) in the rostral migratory stream (RMS). These neuroblasts differentiate in the OB into periglomerular and granular interneurons that can be identified as they permanently retain BrdU incorporated during the S-phase preceding their cell cycle exit. Despite a higher number of proliferative GFAP^+^ cells in the SEZ of SAMP8 mice, DCX^+^ neuroblasts in the RMS (relative to DAPI-stained nuclei: 16.8 ± 2.6 *vs*. a SAMR1 value of 18.2 ± 3.0%, *n* = 3) or BrdU^+^ newly generated neurons in the OB glomerular layer (relative to DAPI-stained nuclei: 0.28 ± 0.01 *vs*. a SAMR1 value of 0.27 ± 0.10%, *n* = 3) were not increased.

To discern among the possible causes that could account for the lack of net effects in neurogenesis, alterations in migration, cell death, and/or proliferation of neuroblasts were investigated. Similar proportions of DCX^+^ cells were found at different anteroposterior levels of the RMS in SAMR1 and SAMP8 mice (data not shown), indicating that neuroblast migration is not affected. In addition, the proportion of DCX^+^ cells that incorporated BrdU injected as a one 1-h pulse was similar in SAMP8 and SAMR1 mice (20.6 ± 4.9 *vs*. 17.3 ± 2.7% respectively, *n* = 4). The number of TUNEL^+^DCX^+^ neuroblasts in the RMS (3110 ± 626 *vs*. a SAMR1 value of 830 ± 496 cells/mm^3^, *n* = 4; *P *<* *0.05) but not of TUNEL^+^GFAP^+^ cells in the SEZ (803 ± 464 *vs*. 839 ± 499 cells/mm^3^ in SAMR1 mice, *n* = 4) was increased in SAMP8 mice, indicating that limited survival of newborn cells counterbalances the increased cycling activity of B1 cells in young SAMP8 mice.

### Increased cycling activity of young SAMP8 NSCs results in their premature senescence *in vitro*

**Fig 3:**
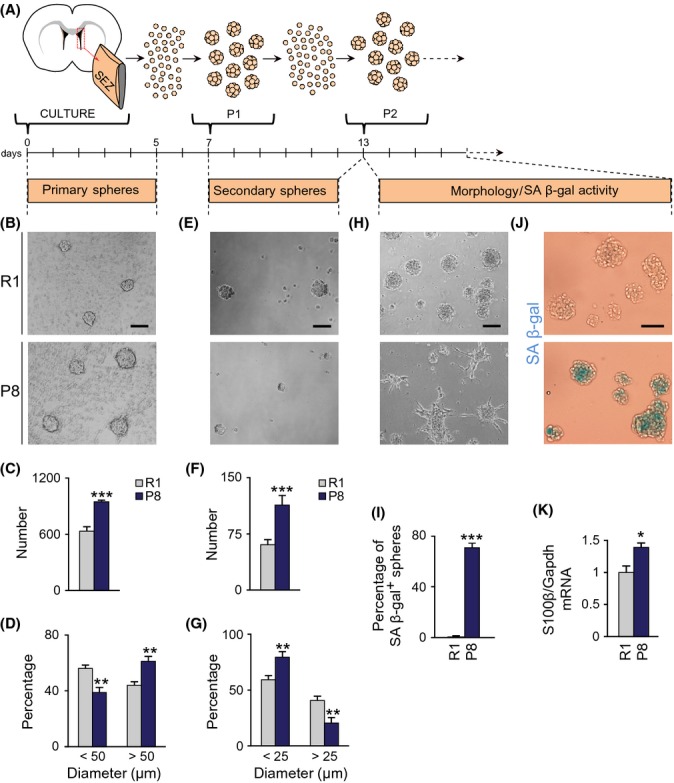
Neurosphere-forming cells from P8 mice are senescent-prone. (A) Schematic diagram of the experimental design. (B) Primary spheres from 2-m R1 and P8 mice in phase contrast. (C) Number of primary spheres per SEZ (R1 *n* = 8, P8 *n* = 8). (D) Size of primary spheres after 5 days (R1 *n* = 8, P8 *n* = 8). (E) Secondary spheres from passage 1. (F) Number of secondary spheres (R1 *n* = 13, P8 *n* = 8). (G) Size of secondary spheres after 6 days (R1 *n* = 13, P8 *n* = 8). (H) R1 and P8 cultures after passage 2. (I) Percentage of spheres that exhibit SA β-gal-positive staining (R1 *n* = 4, P8 *n* = 4). (J) SA β-gal staining in spheres recovered from cultures of secondary neurospheres. (K) Relative expression of S100β mRNA in spheres recovered from cultures of secondary neurospheres (R1 *n* = 5, P8 *n* = 5). Data are shown as mean ± SEM of the indicated number of cultures (*n*) from each strain (*P <* *0.05; **P <* *0.01; ***P <* *0.001). Scale bars: (B) and (H) 100 μm; (E) and (J) 50 μm.

In agreement with the presence of more proliferative GFAP^+^ cells, the SEZ of young SAMP8 mice yielded more primary neurospheres when individual cells were plated with EGF and FGF (Fig.3A–C). Compared to SAMR1, SAMP8 primary neurospheres were also significantly larger in diameter (Fig.3D), had reduced levels of TAP-associated transcription factor Mash1/Ascl mRNA (0.46 ± 0.01 relative to SAMR1 levels, *n* = 3; *P *<* *0.001), and produced more secondary neurospheres when their cells were dissociated and reseeded (passage P1) at very low density (Fig.3E,F). These data indicated that the SEZ of young SAMP8 harbors more NSCs capable of forming neurospheres at young ages and further suggested that BICs are B1 cells modified by the SAM genetic background.

SAMP8 secondary neurospheres were, however, smaller in size after 5 days (Fig.3E,G), incorporated less BrdU (relative to DAPI: 14.7 ± 1.8 *vs*. 22.2 ± 1.5% in SAMR1 cultures; *n* = 7, *P *<* *0.01), and could not be subcultured for another passage. They did not appear to undergo apoptosis (percent caspase 3^+^ cells/DAPI: 1.51 ± 0.14 in SAMP8, *n* = 4, and 1.56 ± 0.24 in SAMR1 cultures, *n* = 3), but adopted a senescent phenotype, characterized by cell enlargement, adhesion to the plastic culture plate, cessation of proliferation, and positivity in the histoenzimatic reaction of the senescence-associated β-galactosidase (SA β-gal) (Fig.3H,J). Some P2 neurospheres could still be observed, but were noticeably smaller and SA β-gal^+^, and had increased levels of S100β mRNA (Fig.3H–K). The strikingly premature senescence of SAMP8 cells was never seen in C57BL/6 or CD1 cells subcultured for many months (Ferron *et al*., 2007). Our data indicated that, although SAMP8 SEZs contain elevated numbers of neurosphere-forming cells, their self-renewal is remarkably reduced.

### Impaired self-renewal of SAMP8 neurosphere-forming cells associates with increased DNA damage and is mediated by p53 and p19

**Fig 4:**
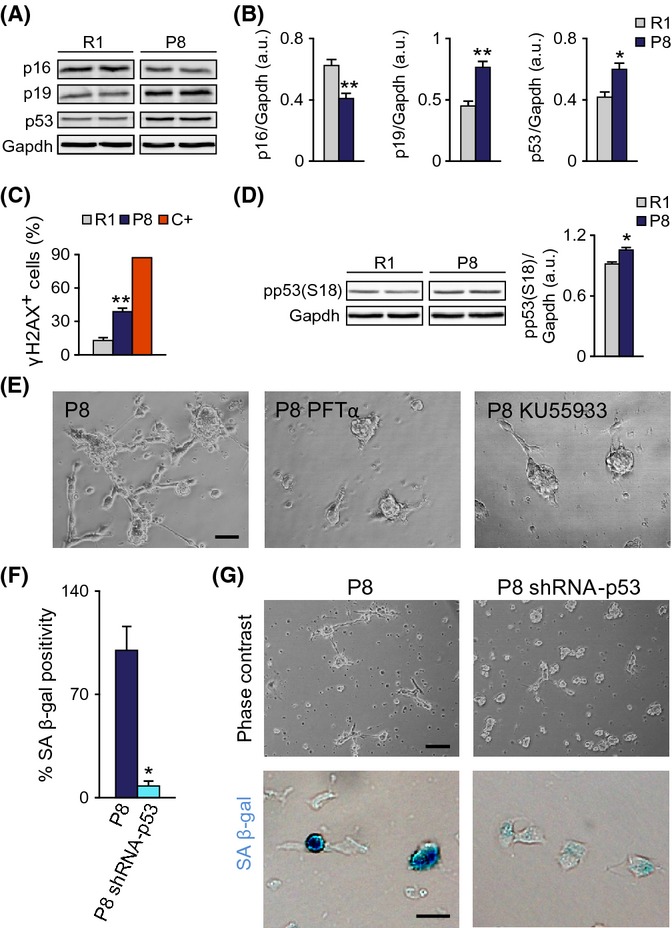
Senescence of P8 cells requires p53. (A) Left: Representative immunoblots for p16, p19, and p53 in P2 neurospheres from 2-m R1 and P8 mice. (B) Densitometric quantification of p16, p19, and p53 relative to Gapdh levels (R1 *n* = 7, P8 *n* = 7). (C) Percentage of cells with γ-H2AX^+^ foci. Positive control (C+) is a doxorubicin-treated (0.5 μg/ml, 6 h) neurosphere culture. (D) *Left*: Representative immunoblot for phospho-p53 in P2 neurospheres from 2-m R1 and P8 mice. *Right*: Densitometric quantification of pp53 relative to Gapdh levels (R1 *n* = 3, P8 *n* = 3). (E) Treatment with 20 μm p53 inhibitor PFTα or 10 μm ATM inhibitor KU55933 prevents the P8 senescent phenotype. (F) SA β-gal labeling of P8 cells infected with a control or with a p53 shRNA-carrying retrovirus (R1 *n* = 4, P8 *n* = 4). (G) Representative images of p53 shRNA and control-infected cultures. *Upper panels*: phase contrast. *Lower panels*: SA β-gal staining. Data are shown as mean ± SEM of the indicated number of cultures (*n*) from each strain (*P <* *0.05; **P <* *0.01). Scale bars: (E), 50 μm; (G, *upper panels*), 100 μm; (G, *lower panels*), 20 μm.

Cellular senescence can be triggered by a number of negative stimuli (telomere erosion, oxidative stress, replicative stress) which activate the DNA damage response (DDR), a signaling pathway in which ATM or ATR kinases block cell cycle progression through stabilization of p53. Activation of certain oncogenes results in derepression of the *Ink4a/Arf* locus in the absence of DNA damage and can also contribute, through increases in p19, in the stabilization of p53 (Gil & Peters, 2006; van Deursen, 2014). In adult neurosphere cultures, loss of proliferation control, that is, as a result of p21 deletion and/or Sox2 overexpression, leads to DNA damage and senescence mediated by increases in p53 and p19, but not p16 (Marqués-Torrejón *et al*., 2013). Likewise, we detected increased levels of p19 and p53, but reduced levels of p16 in SAMP8 relative to SAMR1 cultures by immunoblot (Fig.4A,B), a molecular profile consistent with a DDR.

In SAMP8 neurosphere cultures, we could indeed detect increased proportions of cells with DNA foci immunopositive for the form of histone H2AX phosphorylated in Ser 139 (γ-H2AX), a widespread marker of DNA damage (Fig.4C). Consistent with this, the levels of p53 phosphorylated in Ser18 (pp53) by ATM (Chao *et al*., 2006) were also higher (Fig.4D). Treatment of the cultures with the ATM inhibitor KU55933 or with pifithrin-α (PFTα), a water-soluble inhibitor of p53 activity (Komarov *et al*., 1999), prevented the senescent phenotype (Fig.4E). Senescence was also partly prevented by retroviral delivery of a specific shRNA (Marqués-Torrejón *et al*., 2013) which lowered p53 to 0.68 ± 0.01 (*n* = 3) of the levels in control cultures (Fig.4F,G). These data indicated potential activation of the p53 checkpoint in response to DNA damage in SAMP8 cells.

### Changes in chromatin acetylation regulate neurosphere proliferation and senescence state through the Arf-p53 axis

**Fig 5:**
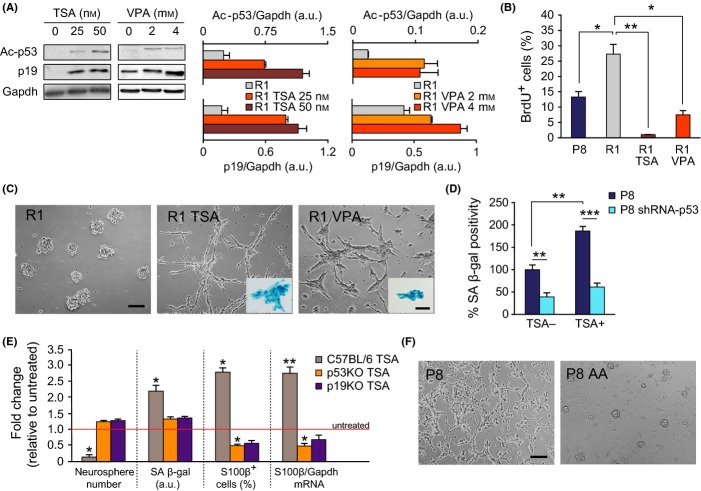
Inhibition of HDACs in neurospheres induces senescence. (A) Left: Representative immunoblot for Ac-p53 and p19 in R1 neurospheres treated with TSA or VPA. *Right*: Densitometric quantification reveals a TSA and VPA dose-dependent increase in Ac-p53 and p19 protein levels, relative to Gapdh (in arbitrary units, a.u.). (B) Percentage of BrdU-positive cells in P8, R1, and R1 TSA- or VPA-treated neurospheres. (C) Secondary spheres from R1 mice treated with vehicle (DMSO), 50 nm TSA, or 4 mm VPA. *Insets*: TSA- and VPA-treated R1 neurospheres exhibit SA β-gal staining. (D) SA β-gal labeling of P8 cells infected with control or p53 shRNA-carrying retroviruses and treated with DMSO or 50 nm TSA (R1 *n* = 5, P8 *n* = 5). (E) Fold changes in the number of neurospheres, of SA β-gal^+^ labeling, in the number of S100β^+^ cells, and the level of S100β mRNA in TSA-treated relative to untreated cultures in C57BL/6 wild-type and p53 or p19 mutant mice. (F) Senescent phenotype of P8 cultures is rescued by treatment with 50 μm anacardic acid (AA). Data are shown as mean ± SEM of 3 independent cultures from each strain or treatment (*P <* *0.05; **P <* *0.01). Scale bars: (C) and (F), 100 μm; (C, *insets*), 40 μm.

Treatment of normal MEFs with trichostatin A (TSA), a natural compound that interferes with the activity of HDACs classes I and II, but not class III (Willis-Martinez *et al*., 2010), leads to increases in p19, together with reductions in p16, and induces senescence (Matheu *et al*., 2005; Simboeck *et al*., 2011). Treatment with HDAC inhibitors also increases acetylation of other substrates, including p53 which then becomes protected from degradation and increases its activity (Luo *et al*., 2000). A 3-day treatment of SAMR1 cells with TSA at 25 or 50 nm raised the levels of acetylated p53 on Lys 379 (ac-p53) and induced the accumulation of p19 in a concentration-dependent manner (Fig.5A). The same results were found with 2 or 4 mm valproic acid (VPA), which also inhibits HDACs by binding to their catalytic center (Göttlicher *et al*., 2001) (Fig.5A). Inhibition of the HDAC activity in SAMR1 primary cultures with either TSA or VPA resulted in reduced BrdU incorporation and drove the cells into a SA β-gal^+^ morphological state similar to that exhibited by SAMP8 cells in basal conditions (Fig.5B,C).

Knockdown of p53 in SAMP8 cells reduced senescence even in the presence of TSA (Fig.5D). Although this result could indicate that increases in p19 act in concert with p53 to trigger cell cycle arrest, we could not dissociate the effect from a DDR-dependent p53 action. We, therefore, decided to explore the effect of HDAC inhibition in NSCs derived from normal mouse strains at passages when DNA damage is not observed (Porlan *et al*., 2013). Cells in primary cultures of adult C57BL/6 mice also entered senescence upon TSA treatment (not shown) as also reported for cultures derived from postnatal day 8 SEZs (Foti *et al*., 2013). At passages 4–7, the same treatment did not cause attachment of the cells to the dish, but resulted in reduced formation of neurospheres together with an increment in SA β-gal^+^ cells (Fig.5E). TSA treatment also yielded neurospheres with elevated levels of S100β mRNA in which more S100β^+^ cells could be found (Fig.5E). TSA effects were not observed in p53- or p19-deficient cultures treated in the same way (Fig.5E), indicating that upregulation of p19 and/or p53 can result in senescence in the absence of DNA damage.

Our results strongly suggested that HDAC activity is required for the maintenance of self-renewal. As HATs counteract the actions of HDACs, we treated SAMP8 neurosphere cultures with 50 μm of the specific inhibitor anacardic acid (AA). The treatment rescued the senescent phenotype of DNA-damaged SAMP8 P2 neurosphere cells (Fig.5F) without affecting SAMR1 cells (not shown), suggesting that the senescent state observed in SAMP8 cultures could be dependent on the epigenetic deregulation of the *Ink4a/Arf* locus.

### HDAC inhibition *in vivo* reproduces the phenotype of young SAMP8 mice

**Fig 6:**
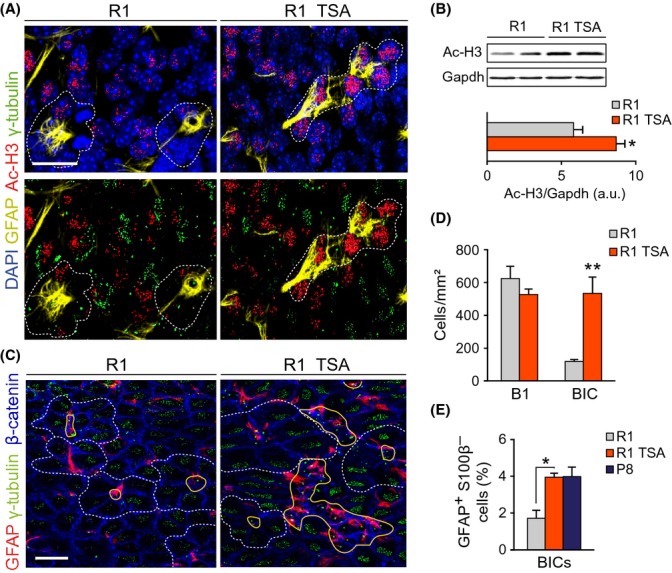
Treatment of R1 mice with TSA mimics the altered SEZ features of young P8 mice. (A) Ac-H3, GFAP, and γ-tubulin in a whole-mount preparation of a 2-m R1 mouse treated with TSA (R1 TSA) or vehicle (R1). (B) *Up*: representative immunoblot for Ac-H3 in SEZ homogenates from TSA or vehicle-treated 2-m R1 mice. *Down*: densitometric quantification of Ac-H3 relative to Gapdh levels (R1 *n* = 3, P8 *n* = 3). (C) GFAP, γ-tubulin, and β-catenin in SEZ whole-mounts of TSA-treated and untreated R1 mice. Note the presence of clusters of BICs in the TSA-treated animal. (D) Numbers of B1 and BICs in R1 mice treated or not with TSA (R1 *n* = 4, R1 + TSA *n* = 3). (E) Percentage of GFAP^+^S100β^−^ BICs relative to total cells (R1 *n* = 3, P8 *n* = 3). Pinwheels are delineated by dashed white lines, and B1 cells are delineated by solid yellow lines. Data are shown as mean ± SEM of the indicated number of independent mice (*n*) from each strain (*P <* *0.05; **P <* *0.01). Scale bars: (A) 15 μm, (C) 30 μm.

We next wondered whether epigenetic alterations due to deficient HDAC activity could also result in alterations of the SEZ similar to those observed in young SAMP8 mice. To investigate this possibility, we injected young adult SAMR1 mice with either TSA at 1 mg/kg of body weight or with the DMSO vehicle twice every day during 3 days and analyzed the lateral ventricle wall. The treatment resulted in increased levels of histone 3 acetylated in Lys 9 (ac-H3), as determined by immunohistochemistry and immunoblot (1.5 ± 0.1-fold increase with respect to vehicle injected mice; Fig.6A,B).

Despite unchanged numbers of pinwheels, a raise in the occurrence of uniciliated GFAP^+^ cells integrated within the ependyma, similar to that previously observed in SAMP8 mice, was induced in SAMR1 mice treated with TSA (Fig.6C,D). Moreover, counts in SEZ sections indicated that the treatment resulted in a higher proportion of GFAP^+^S100β^−^ cells integrated in the ependymal layer (Fig.6E). Together with the restoration of the phenotype of SAMP8 neurospheres *in vitro* by HAT inhibition, this observation suggests that epigenetic changes may, at least partly, underlie the changes observed in the SEZ of SAMP8 mice.

## Discussion

Aging is a negative regulator of adult neurogenesis, and in turn, decreased neurogenesis is considered a contributor to age-associated cognitive and olfactory declines. Yet, the mechanisms underlying impaired neurogenesis in the aging brain are poorly understood. We have analyzed B1-NSC behavior in SAMP8 mice with the aim of identifying molecular mechanisms that may impinge in their aging process. Our data support a model in which epigenetic derepression of tumor suppressor p19 drives accelerated senescence and loss of subependymal NSCs with a SAMP8 genetic background. Interestingly, these NSCs exhibit a transient increase in proliferative activity before exhaustion *in vivo* that can be reproduced by HDAC inhibition.

We find that the SEZ of young SAMP8 and SAMR1 mice contains fewer B1-NSCs than other inbred strains, suggesting a significant effect of the SAM genetic background in B1-cell generation and/or survival. Increased numbers of multiciliated GFAP^+^ cells in SAMP8 and SAMR1 mice could indicate that B1 cells are not properly maintained in SAM strains, but are more prone to become ependymal cells, a fate that is associated with older ages in other inbred strains (Luo *et al*., 2008). However, other reports using BrdU retention do not support B1-cell transformation into ependymocytes (Capilla-Gonzalez *et al*., 2014). Exploring this possibility would require lineage tracing analysis as ependymal cells are no longer detectable by GFAP immunohistochemistry. Nevertheless, SAMP8 mice loose B1 cells more rapidly, resulting in significant differences in the number of pinwheels between SAMR1 and SAMP8 at 10-m.

Accelerated loss of B1 cells in SAMP8 mice is preceded by abnormally located B1-like cells (BICs) which are proliferative and most likely form neurospheres. This is in line with previously reported increases in BrdU incorporation in the SEZ and the SGZ of these mice (Gang *et al*., 2011; Díaz-Moreno *et al*., 2013). These reports together with data presented here also indicate that increased proliferation is not productive due to reduced viability of the generated neurons. Interestingly, HDAC inhibition reduces neurosphere formation and postnatal neurogenesis *in vivo* (Zhou *et al*., 2011; Foti *et al*., 2013), further suggesting that the increase in BICs that we observe after TSA treatment and in SAMP8 mice is also likely to be nonproductive.

Expression of the *Ink4a/Arf* locus is silent during embryogenesis, but is gradually upregulated with aging (Zindy *et al*., 1997; Krishnamurthy *et al*., 2004). One or both proteins encoded by this locus are induced during senescence depending on triggers and cellular context, with notable species-specific differences, that is, murine cells being more dependent on p19 that human cells (Bruggeman *et al*., 2005; van Deursen, 2014). Protein p16 appears to limit NSC potential during physiological aging (Molofsky *et al*., 2006), but we find increased levels of p19 specifically in SAMP8 neurospheres. Thus, the net effect of bidirectional changes in tumor suppressor protein expression remains to be elucidated. The p19–p53 pathway not only regulates NSC self-renewal and proliferation in the postnatal and adult brains (Bruggeman *et al*., 2005; Nagao *et al*., 2008; Ahlenius *et al*., 2009), but also their responsiveness to gliogenic signals (Nagao *et al*., 2008). An increase in p19–p53 could couple senescence to a gliogenic program that might promote terminal differentiation of BICs into non-neurogenic S100β^+^ cells. Furthermore, we have recently shown that loss of proliferation control and/or increased replicative stress leads to higher levels of p19, but not p16 and results in cell senescence in neurosphere cultures and higher proportions of S100β^+^ cells *in vivo* (Marqués-Torrejón *et al*., 2013). Although p53 and p21 control at least in part the proliferation of NSCs (Medrano *et al*., 2009; Marqués-Torrejón *et al*., 2013; Porlan *et al*., 2013), we have failed to detect altered expression of p21 in the SEZ from SAMP8 mice (data not shown).

Our results indicate a regulation of the *Ink4a/Arf* locus by acetylation. HDAC inhibition reduces proliferation of adult hippocampal progenitors and promotes their neuronal differentiation through the upregulation of the neurogenic basic helix-loop-helix transcription factor NeuroD (Hsieh *et al*., 2004). In these progenitor cells, the orphan nuclear receptor TLX recruits HDACs to repress transcription of the cell cycle inhibitor p21 and of the tumor suppressor PTEN and deregulation of p21 has been shown to be important for the antiproliferative effect of HDAC inhibitors, although p19 was not tested (Sun *et al*., 2007). The relationship of PcG complexes with *Ink4a/Arf* expression is also a provocative possibility given the ability of these complexes to create heritable epigenetic marks. PcG gene product Bmi1 deficiency in mice is associated with premature senescence and loss of NSC self-renewal, which can in large part be rescued by repressing the expression of p16 and p19 (Bruggeman *et al*., 2005; Molofsky *et al*., 2005; Nishino *et al*., 2008).

The underlying genes responsible for accelerated senescence and pathological changes in SAM strains remain largely unidentified. A recent whole-exome sequencing of various SAM strains has revealed the existence of some SAMP-shared and SAMP8-specific single nucleotide variations (Tanisawa *et al*., 2013). Two of these mutations are found in DNA repair genes *Ogg1* and *Mbd4* and thus may be directly involved in the susceptibility to diseases via defects in the DDR. A third specific mutation is present in the *Aifm3* gene, encoding the apoptosis-inducing factor mitochondrion-associated protein 3, which may contribute to mitochondrial dysfunction in SAMP8 mice as it induces apoptosis *in vitro* and has an oxidoreductase domain that might play a role in the respiratory chain (Xie *et al*., 2005).

Our data suggest that the NSC loss that takes place during physiological aging in mice is dramatically accelerated in a SAMP8 background and provides a system in which to analyze the mechanisms underlying exhaustion of stem cell pools *in vivo*. Moreover, the involvement of p19 in neurogenic senescence suggests the possibility that neurogenic dysfunction in the elderly could be prevented by pharmacological treatment.

## Experimental procedures

### Animals

Adult male mice of SAMP8 and SAMR1 (Jackson Laboratories, (Charles River, Barcelona, Spain)), p53 (Marqués-Torrejón *et al*., 2013) and p19 knockout (Kamijo *et al*., 1997), C57BL/6, and CD1 strains were handled in accordance with Spanish RD1201/2005 guidelines, following protocols approved by the institutional committees. BrdU (50 mg/kg body weight in saline solution; Sigma) was intraperitoneally injected, either seven times in a 12-h period (one injection every 2 h) 30 days before perfusion or as a single dose 1 h before perfusion. TSA (1 mg/kg body weight; Sigma) or DMSO was administered by intraperitoneal injection, at one dose every 12 h during 3 days, and the SEZs were dissected 1 h after the last injection.

### Histological analyses

Mice were anesthetized (225 mg/kg of ketamine and 3 mg/kg of medetomidine) and transcardially perfused with 4% paraformaldehyde in 0.1 m phosphate buffer pH 7.4 (PB). The brains were dissected, postfixed by immersion in the same fixative overnight (o/n), and serially sectioned into 25-μm coronal sections using a Leica VT 1000S vibratome. For whole-mounts, the SEZs were processed as described (Mirzadeh *et al*., 2008). Sections and whole-mounts were blocked using 10% FBS and 0.2% Triton X-100 in PB. For BrdU immunodetection, sections were first treated with 2N HCl for 15 min at 37 °C. Samples were incubated in rabbit anti-Ki67 (1:150, Abcam, Cambridge, England), S100β (1:150, Dako), β-catenin (1:100, Cell Signaling), or ac-H3 (1:10 000, Sigma); rat anti-BrdU (1:600, Abcam); chicken anti-GFAP (1:400, Millipore, Madrid, Spain); goat anti-Sox2 (1:50, RyD), DCX (1:100, Santa Cruz); mouse anti-γ-tubulin (1:600, Santa Cruz, Heidelberg, Germany); or acetylated α-tubulin (1:500, Sigma) antibodies in blocking buffer, o/n at 4 °C. After several PB washes, the sections were incubated for 1 h at room temperature (RT) with DyLight 549, DyLight 488, or Cy3-conjugated appropriate secondary antibodies (1:600 to 1:1500; Jackson ImmunoResearch Laboratories, Suffolk, England). Samples were stained with DAPI (Sigma) at 1 μg/mL and mounted with Fluorsave (Calbiochem, Madrid, Spain). Confocal planes along the rostrocaudal axis or z images of the entire dorsal aspect of SEZ whole-mounts were captured using a Fluoview FV10i (Olympus) confocal microscope and analyzed. Apoptotic cells were detected by terminal deoxynucleotidyl transferase-mediated dUTP nick-end labeling (TUNEL) using ApopTag *In Situ* Apoptosis Detection Kit (Millipore) according to the manufacturer protocol. For scanning electron microscopy, whole-mounts fixed as before were dried with the critical point method and shadowed with gold–palladium and examined with a Hitachi scanning electron microscope.

### NSC culture, transduction, immunocytochemistry, and SA β-gal staining

Methods for adult NSC culture and self-renewal assessment have been described (Ferron *et al*., 2007). TSA, AA, VPA, KU55933, and PFTα were from Sigma. Vectors containing p53 shRNAs, methods used for retrovirus production, and NSC transduction have been described (Marqués-Torrejón *et al*., 2013). In brief, single cells obtained from passage 1 (P1) neurospheres were infected o/n by incubation with diluted (1:5) supernatants of shRNA retroviruses produced in Plat-E cells; after 5 days, the clones were dissociated and single cells were adhered for 10 min to a Matrigel substrate and stained. The Senescence Cells Histochemical Staining Kit (Sigma) was used following manufacturer's instructions. Representative images were obtained in a Nikon Eclipse E800 microscope and positivity was quantified using imagej 1,49i software (NIH, USA). Briefly, cyan pixels were segmented by color threshold and their total intensity was divided by the number of cells in each image. For immunocytochemistry, incubation with antibodies for mouse anti-γH2AX (1:100, Millipore), rat anti-BrdU (1:600, Abcam), and rabbit anti-S100β (1:1200, Dako) was performed on Matrigel-attached freshly dissociated NSCs as described (Ferron *et al*., 2007). Secondary antibodies and DAPI counterstaining were as for histological analyses. Pictures of random fields in each condition were taken on the InCell Analyzer 2000 (GE Healthcare, Valencia, Spain), and positive cells were scored using the InCell investigator 1.6 software.

### Protein isolation/Western blotting

Neurospheres were homogenized in lysis buffer (20 mm PBS, 150 mm NaCl, 5 mm EDTA, 1% Triton X-100, 2,5 mm sodium orthovanadate, 20 mm sodium fluoride, 2 mm phenyl-methane-sulfonylfluoride and 1X Complete Mini tablet (Roche) for 20 min and centrifuged at 20 000 *g* for 15 min at 4 °C. Protein concentration was determined by Pierce BCA Protein assay kit (Thermo Scientific, Madrid, Spain). Protein extracts (50–70 μg) were denatured in Laemmli's sample buffer containing β-mercaptoethanol, sodium dodecyl sulfate (SDS), and bromophenol blue, separated by 10% SDS–polyacrylamide gel electrophoresis and transferred to a nitrocellulose membrane (Sigma). The membranes were blocked 1 h at room temperature with 5% nonfat dry milk in Tris-buffered saline supplied with 0.1% Tween 20 (TBS-T) and incubated with rabbit anti-p53 (1:500, Novocastra), ac-p53 (1:1000, Cell Signaling), ac-H3 (1:10 000, Sigma), p16 (1:200, Santa Cruz) or pp53 (S18) (1:500, RyD), rat anti-p19 (1:1000, Abcam), or mouse anti-Gapdh (1:1500, Millipore) antibodies in blocking buffer, o/n at 4 °C. After extensive washing with TBS-T, membranes were incubated for 1 h at RT with horseradish peroxidase-conjugated goat antibodies to mouse (1:2000, Dako), rabbit (1:5000, Amersham), or rat (1:1000, Amersham) IgGs in blocking buffer. Peroxidase activity was detected with Western Lightning Plus reagents (Perkin Elmer) according to supplier recommendations.

### RNA Isolation and qRT–PCR analysis

Total RNA was extracted using TRIzol and cDNA synthesized using PrimeScript RT reagent kit (Takara). cDNA products were amplified on a StepOnePlus Real-Time PCR System (Applied Biosystems) using Premix Ex Taq (Probe qPCR) (Takara). Taqman probes for mouse S100β (Mm00485897_m1) and Gapdh (Mm99999915_g1) were purchased from Applied Biosystems.

### Statistics

Data are presented as the mean ± SEM of a number (*n*) of cultures or independent mice *per* experimental condition. Statistical significance was determined by two-tailed Student's t-tests or two-way anova with *post hoc* analysis by Bonferroni's test. Significance was set at *P <* *0.05, **P <* *0.01, or ***P <* *0.001. Statistical analyses were carried out using SPSS V. 17 (2008 SPSS Inc., Chicago, IL, USA) or GraphPad Prism (2007 GraphPad Software Inc.).
